# Impacts of Roasting Intensity and Cultivar on Date Seed Beverage Quality Traits and Volatile Compounds Using Digital Technologies

**DOI:** 10.3390/foods14223902

**Published:** 2025-11-14

**Authors:** Linghong Shi, Hanjing Wu, Kashif Ghafoor, Claudia Gonzalez Viejo, Sigfredo Fuentes, Farhad Ahmadi, Hafiz A. R. Suleria

**Affiliations:** 1 School of Agriculture, Food and Ecosystem Sciences, Faculty of Science, The University of Melbourne, Parkville, VIC 3010, Australia; linghongs@student.unimelb.edu.au (L.S.); hanjing.wu@student.unimelb.edu.au (H.W.); kashif.ghafoor@unimelb.edu.au (K.G.); farhad.ahmadi@unimelb.edu.au (F.A.); 2 Digital Agriculture, Food and Wine Research Group, School of Agriculture, Food and Ecosystem Sciences, Faculty of Science, The University of Melbourne, Parkville, VIC 3010, Australia; cgonzalez2@unimelb.edu.au (C.G.V.); sigfredo.fuentes@unimelb.edu.au (S.F.); 3 Tecnologico de Monterrey, School of Engineering and Science, Ave. Eugenio Garza Sada 2501, Monterrey 64849, Mexico

**Keywords:** date seed, coffee alternative, volatile compounds, non-destructive sensing, machine learning

## Abstract

Roasting intensity and cultivar shape the physicochemical composition and sensory characteristics of date seed-based coffee alternatives. This study evaluated quality traits among eight date seed cultivars (Zahidi, Medjool, Deglet nour, Thoory, Halawi, Barhee, Khadrawy, Bau Strami) roasted at three intensities (light: 180 °C; medium: 200 °C; dark: 220 °C) using digital technologies, including near-infrared spectroscopy (NIR), electronic nose (e-nose), and headspace solid-phase microextraction gas chromatography-mass spectrometry (HS-SPME-GC-MS), supported by machine learning (ML) modeling. NIR spectra showed distinct chemical fingerprints for date seed powders and beverages, with key absorption bands from 1673–2396 nm and 1720–1927/2238–2396 nm, respectively. E-nose outputs showed higher volatile emissions in dark-roasted samples, particularly for ethanol and NH_3_. GC-MS identified 25 volatile compounds, mainly pyrazines and furanic compounds. Pyrazine concentration was greatest in Bau Strami and Medjool cultivars, whereas Halawi and Thoory cultivars had greater content of furfural. Two ML classification models achieved high accuracy in classifying cultivars (NIR inputs: 99%; e-nose inputs: 98%) and roasting levels, while regression models (NIR inputs: *R* = 0.88; e-nose inputs: *R* = 0.90) effectively predicted volatile aromatic compounds obtained using GC-MS. Dark roasting resulted in a significant pH reduction and intensified browning, with furfural persisting as a stable aroma contributor. These findings highlight the potential of date seeds as a coffee alternative, with roasting level and cultivar selection influencing flavor profiles. The findings also demonstrate the utility of digital sensing technologies as an efficient, low-cost tool for rapid quality assessment and process optimization in the development of novel beverages.

## 1. Introduction

The date palm (*Phoenix dactylifera* L.) is a historically important crop, widely cultivated in arid and semi-arid regions, with more than 2000 known cultivars worldwide, including prominent commercial ones such as Medjool, Barhee, and Deglet nour [1,2]. Although the fruit pulp is well known for its high carbohydrate content and essential minerals, the nutritional potential of the date seed is often underestimated. Previous studies have shown that date seeds contain comparable carbohydrate levels and, in some cases, significantly higher concentrations of trace minerals than the pulp. Moreover, they are rich in polyphenolic compounds and show strong antioxidant activity, which may contribute to health-promoting effects [2].

Despite these benefits, date seeds are typically treated as by-products and are usually discarded. In certain regions, particularly in Saudi Arabia and the United Arab Emirates, there is a traditional practice of roasting, grinding, and brewing date seeds to produce a caffeine-free, coffee-like beverage [3,4]. This beverage has gained interest as a coffee alternative due to its similar aroma, appearance, and texture, but without the stimulant effects of caffeine. As with conventional coffee, roasting plays an important role with two main reasons. Firstly, from a technological and safety perspective, roasting reduces moisture content, inactivates spoilage-related enzymes, and extends shelf life. Second, and more importantly for sensory quality, it promotes Maillard reactions, pyrolysis, and caramelization, which would generate the characteristic color, roasted flavor, and rich aroma that enhance consumer appeal [5,6].

While the roasting process of coffee has been studied in detail, leading to the identification of key volatile markers like furans, pyrazines, and pyrroles that define its sensory quality, research on date seed roasting still remains limited. A few existing studies have detected Maillard-derived volatiles in roasted date seeds similar to those in roasted coffee [7,8]. However, these studies often lack a systematic evaluation of how roasting parameters such as temperature and duration, as well as the intrinsic compositional differences among date cultivars might potentially influence volatile formation and sensory quality.

Despite growing interest in utilizing date seeds for the production of seed-based beverages, current studies remain limited and largely non-systematic, particularly concerning the combined influence of date seed cultivar and roasting intensity on beverage properties. The effects of these factors on the physicochemical characteristics, aroma compound development, and overall quality of date seed beverages remain poorly characterized. Therefore, the aim of this study was to investigate the influence of roasting intensity across eight date seed cultivars on quality traits of brewed date seed beverages. A combination of digital technologies, including NIR spectroscopy, e-nose, and headspace solid-phase microextraction gas chromatography-mass spectrometry (HS-SPME-GC-MS), was used to evaluate volatile profiles, chemical attributes, and classification performance. This research aims to contribute to a better understanding of how roasting and cultivar selection affect the development of high-quality, coffee-like beverages from date seeds.

## 2. Materials and Methods

### 2.1. Sample Preparation

Eight date cultivars at the *Tamar* ripening stage (Zahidi, Medjool, Deglet nour, Thoory, Halawi, Barhee, Khadrawy, and Bau Strami) were used in this study, obtained from the Desert Fruit Company (Alice Springs, Northern Territory, Australia). Date fruits were processed following the protocol described by Shi et al. [9]. First, the flesh and seeds were separated, and for each sample, 30 g of seeds were divided into three parts (10 g each) then roasted at three roasting levels (180 °C, 10 min; 200 °C, 10 min; 220 °C, 10 min) in a Qualtex Solidstat oven (Model IW24S, Qualtex Inc., Brisbane, Australia), based on the method of Wu et al. [10] with modifications. Roasting temperatures were monitored via the oven’s built-in thermometer, and roasting levels were quantified using the Agtron Scale as a standard reference. After roasting, the date seeds were ground into a powder with an 800 g capacity grinder (Laobenhang, model 400Y, Yongkang, Zhejiang, China) and sequentially sieved through a dual-layer mesh screen (upper 80-mesh and lower 100-mesh) to ensure uniform particle size distribution. For brewing, 2.5 g of the ground date seed powder was extracted using a Breville Creatista^®^ Plus espresso machine (Breville Pty Ltd., Sydney, Australia) in Espresso mode, dispensing a constant volume of 25 mL at 78 °C. Each extraction was performed in triplicate. In total, 24 liquid samples derived from the different roasting conditions and cultivars (Table 1) were prepared for subsequent analysis.

### 2.2. Physicochemical Characterization of Date Seed Beverage Brew

#### 2.2.1. pH Measurement

The pH of the date seed brew was assessed in triplicate with a LAQUAtwin Compact pH meter (HORIBA’s, Kyoto, Japan). The meter was calibrated prior to use with three standard buffer solutions at pH 4.0, 7.0, and 9.0.

#### 2.2.2. Color Measurement

The color of date seed beverage samples with different roasting levels was measured in triplicate using a hand-held colorimeter NIX (Nix Pro Color Sensor™, Nix Sensor Ltd., Ontario, CA, USA). For powdered samples, 5 g of ground date seed was evenly distributed in a transparent plastic dish to create a uniform surface, and color measurements were taken at random positions across the sample area. For beverage samples, the colorimeter was immersed in 2 mL of the sample without touching the bottom of the container, and readings were taken against a pure white background. The background color was measured separately. The colorimetric coordinates (L*, a*, b*) were recorded and analyzed. The color difference (ΔE) was calculated using the following equation:

(1) $$\Delta E=\sqrt{{\left(L_{a1}^{*}-L_{a2}^{*}\right)}^{2}+{\left(a_{a1}^{*}-a_{a2}^{*}\right)}^{2}+{\left(b_{a1}^{*}-b_{a2}^{*}\right)}^{2}}$$

where *a*_1_ is the average value of the first roasting level of date seed beverages, and *a*_2_ is the average value of the second roasting level of date seed beverages.

### 2.3. Near-Infrared Spectroscopy (NIR) Analysis

A handheld near-infrared (NIR) spectrometer, the microPHAZIR™ (RX Analyzer; Thermo Fisher Scientific, Waltham, MA, USA), was employed to assess both date seed powder and beverage samples, following the method described by Gonzalez Viejo, et al. [11]. The device operated across a spectral range of 1596–2396 nm. Measurements were performed in triplicate, with three readings per replicate (*n* = 9 measurements per sample). For measurement, a 7.0 cm qualitative grade 3 Whatman^®^ filter paper (Whatman plc, Maidstone, UK) was soaked in each date seed beverage sample. To minimize interference from ambient light, a white background was placed above the sample during analysis. The absorbance readings of the dry, blank filter paper were deducted from those of the wet, sample-loaded filter paper to isolate the spectral signature of the date seed beverage, following the method of Gonzalez Viejo, Fuentes, Torrico, Howell and Dunshea [11]. Spectral data were processed using The Unscrambler X (version 10.3; CAMO Software, Oslo, Norway), applying Savitzky–Golay filtering to obtain first derivative spectra for subsequent analysis.

### 2.4. E-Nose and Data Extraction

To evaluate the volatile compounds in date seed beverage samples, a cost-effective and portable e-nose designed by the University of Melbourne’s Digital Agriculture Food and Wine Group (DAFW-UoM) was employed, as described by Gonzalez, et al. [12,13,14]. The device comprised nine different gas sensors: (i) MQ3: Alcohol (ethanol), (ii) MQ4: Methane (CH_4_), (iii) MQ7: Carbon monoxide (CO), (iv) MQ8: Hydrogen (H_2_), (v) MQ135: NH_3_/alcohol/benzene, (vi) MQ136: Hydrogen sulfide (H_2_S), (vii) MQ137: NH_3_, (viii) MQ138: Benzene/alcohol/NH_3_, and (ix) MG811: Carbon dioxide (CO_2_). Prior to measurement, a 30 s baseline reading was recorded, followed by a 1 min exposure of the samples to the sensors. Signal outputs were analyzed using a custom MATLAB^®^ R2021a (MathWorks, Inc., Natick, MA, USA) script developed by DAFW-UoM. The software displayed sensor signal curves to identify the most stable region, which was then divided into 10 equal segments to calculate mean values for each section automatically [13].

### 2.5. Identification and Quantification of Volatiles by HS-SPME-GC-MS

The volatile composition of date seed beverage samples was characterized using an optimized headspace solid-phase microextraction gas chromatography-mass spectrometry (HS-SPME-GC-MS) protocol based on methodologies from Gonzalez Viejo, Tongson and Fuentes [13] and Wu et al. [15]. The analytical system consisted of three integrated components: (1) an Agilent 6850 Series II gas chromatograph (Agilent Technologies, Santa Clara, CA, USA), (2) a PAL RSI I20 automated headspace sampler (CTC Analytics AG, Zwingen, Switzerland), (3) an Agilent 5973 mass selective detector (Agilent Technologies, Santa Clara, CA, USA). Separation was achieved using an Agilent DB-Wax capillary column (30 m × 0.25 mm ID, 0.25 μm film thickness) paired with a 65 μm PDMS/DVB extraction fiber (Sigma-Aldrich, Sydney, Australia).

Helium served as the mobile phase at a constant head pressure of 60 kPa. Sample preparation involved 15 min thermal equilibration at 60 °C before volatile collection. The extraction process comprised 15 min adsorption followed by 6 min thermal desorption. The temperature gradient program initiated at 40 °C (5 min hold), then ramped at 5 °C/min to 190 °C (8 min), followed by a 10 °C/min increase to 220 °C (10 min final hold). Mass spectra were acquired in SCAN mode (35–350 *m*/*z* range) with a 2 min solvent delay.

A 2.5 g portion of date seed powder was mixed with 100 μL of 100 mg/L 3-heptanone as an internal standard and transferred into vials for analysis. The mixture was injected following the temperature gradient program previously described. The linear retention index (LRI) of each volatile compound was calculated using a series of n-alkane standards (C_7_–C_30_), according to the following equation:

$$\mathrm{LRI}\;\left(\mathrm{target}\;\mathrm{compound}\right)=100\;\times \left(\frac{RT_{X}\;-\;RT_{n}}{RT_{n+1}\;-\;RT_{n}}+n\right)$$

where *RT_x_* is the retention time of the target compound, and *RT_n_* and *RT_n_*_+1_ are the retention times of the n-alkanes eluting immediately before and after the target compound, respectively. The identification of volatile compounds in date seed beverage samples involved comprehensive spectral matching, with linear retention indices (LRIs) validated against reference values in the National Institute of Standards and Technology (NIST; National Institute of Standards and Technology, Gaithersburg, MD, USA) library and mass spectra compared to authentic standards in the NIST Mass Spectral Database (Washington, DC, USA). Following confirmation of compound identity through both LRI and mass spectral data, semi-quantitative analysis was performed by normalizing the peak areas of target compounds against the internal standard. Relative concentrations were subsequently determined using calibration curves constructed from analytical standards of known concentrations.

### 2.6. Statistical Analysis and Machine Learning (ML) Modeling

All results were presented as mean ± standard error (SE) based on three independent replicates, with values corrected by subtracting the corresponding blank or control measurements. Statistical analyses were performed using Minitab^®^ 19 (Minitab Inc., Chicago, IL, USA) and GraphPad Prism version 9.0 (GraphPad Software, La Jolla, CA, USA). One-way analysis of variance (ANOVA) was conducted to evaluate differences among sample groups, with statistical significance set at *p* < 0.05. Pairwise comparisons were performed using Tukey’s HSD post hoc test (*α* = 0.05).

**Figure 1:**
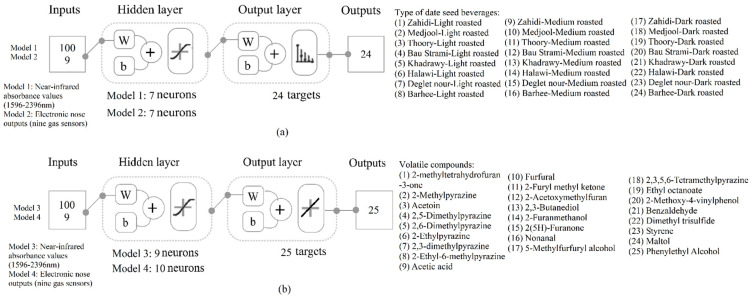
Diagrams of the two-layer feedforward artificial neural network (**a**) classification Models 1 and 2, and (**b**) regression Models 3 and 4, showing the specific inputs, targets, and number of neurons used. Abbreviations: W = weights; b = bias.

Four ML models were constructed using a MATLAB R2019b code developed by the Digital Agriculture Food and Wine group at the University of Melbourne to assess 17 training algorithms and identify the most accurate models with no under- or overfitting in a loop [16] as shown in Figure 1. Models 1 and 2 were developed for classification using the absorbance values obtained from NIR spectra within the range of 1596 and 2396 nm, and e-nose outputs, as inputs, respectively, to predict the cultivar of date seeds and the roasting level of date seed powder. For the NIR analysis, each sample was scanned in triplicate to account for instrumental variability. For the e-nose analysis, 10 consecutive measurements were collected from each sample after the gas sensors had stabilized. All models were constructed using the Bayesian regularization algorithm. Samples were divided using a random data division (dividerand) algorithm, with 70% allocated for the training stage and 30% for testing, based on a performance algorithm that minimized the mean squared error (MSE).

## 3. Results and Discussion

### 3.1. Physicochemical Properties

#### 3.1.1. pH

The pH values of date seed beverages produced from eight cultivars of date seeds at three roasting levels are shown in Table 2. Overall, the roasting intensity significantly influenced the pH of the beverages. Beverages made from the Zahidi cultivar consistently exhibited the highest pH values across all roasting levels. In contrast, beverages produced from the Khadrawy cultivar showed the lowest pH values in each corresponding category. In addition, lightly roasted date seed beverages had significantly higher pH values (*p* < 0.05) compared to medium and dark roasted counterparts, with an overall average pH of 5.95. In some cases, the difference in pH between medium and dark roasts was not significant (*p* > 0.05); for example, in the Khadrawy cultivar (medium roasted: 3.79; dark roasted: 3.39). Nonetheless, a general trend of decreasing pH with increasing roasting intensity was observed. This reduction in pH may be attributed to the formation and subsequent dissolution of acidic compounds, such as pyruvic acid, during the caramelization process that occurs during roasting [17]. These findings are consistent with those reported by Fikry, Yusof, Al-Awaadh, Rahman, Chin, Mousa and Chang [8], who investigated the impact of roasting on the Sukkari date seed cultivar.

#### 3.1.2. Color Measurement

The color of date seed beverages, a key quality attribute influencing consumer acceptance, was significantly affected by both roasting level and cultivar. The color values (L*, a*, and b*) of all the beverage samples and the color difference (ΔE) between the average value of the date seed beverages at different roasting levels are shown in Table 3. In this study, the roasting intensity had a significant impact on color, both in the date seed powder and the resulting beverages. A substantial color difference was observed between light and medium roasting levels (powder: ΔE = 20.62; beverage: ΔE = 18.51). In contrast, the differences between medium and dark roasting were relatively minor (powder: ΔE = 6.08; beverage: ΔE = 2.86), suggesting that beyond a certain threshold, the incremental effect of increasing temperature on color diminishes. In general, L* values decreased with increasing roasting intensity, indicating a progressive darkening of the samples. Similar trends were also observed for the a* and b* values, which may reflect changes in red-green and yellow-blue chromatic components, respectively. This has been previously reported by Babiker et al. [18] and Tsai and Jioe [19] who studied the effect of roasting level on Soukari cultivar date seeds and Coffea Arabica typical from three different origins, respectively. This phenomenon is primarily produced through the Maillard reaction and sugar caramelization reaction during roasting, which is widely observed in coffee and coffee-like beverages. The products generated from these reactions may exhibit functional properties and unique aromas [20].

Variations in color attributes were also evident among different date seed cultivars at specific roasting levels. Among the lightly roasted date seed powders, the Thoory cultivar had the highest L* value (51.9), along with relatively elevated a* and b* values (14.4 and 23.1, respectively), indicating a lighter and more vividly colored appearance. In contrast, the Barhee cultivar showed the lowest values across all three color parameters, suggesting a comparatively darker and less chromatic profile. After medium roasting, the color differences among the various date seed powders became less pronounced. No significant differences (*p* > 0.05) were observed in L*, a*, or b* values among the cultivars at this roasting level. However, with dark roasting, significant variations re-emerged. At this stage, the Bau Strami cultivar had the highest L* value (22.83), indicating a relatively lighter appearance compared to other cultivars. The Medjool cultivar had the highest a* (13.47) and b* (14.97) values, reflecting greater red and yellow color intensities, respectively.

No significant differences in L* values were observed among the beverages prepared from lightly roasted date seeds (*p* > 0.05). However, the Zahidi cultivar exhibited the highest a* value (5.13) among all eight cultivars, suggesting a more pronounced reddish hue in beverages brewed from Zahidi seeds at this roasting level. All lightly roasted beverages displayed negative b* values, indicating a bluish tint. At the medium roasting level, the highest L* value was recorded for the Khadrawy cultivar (54.77), while the lowest was found in the Zahidi cultivar (44.70). Compared to the lightly roasted samples, the average a* value significantly decreased from 3.43 to 1.74, indicating a reduction in red color intensity as roasting temperature increased. Conversely, the b* values increased substantially, with the average b* shifting from −13.43 to 4.33. This shift reflects a perceptible transition in color from bluish in lightly roasted beverages to yellowish in medium-roasted ones.

This trend became more pronounced at the dark roasting level. The average L* (46.77) and a* (1.44) values continued to decrease, but the average b* value increased further to 6.65, giving the beverages a deeper brown appearance. Such color development agrees with expectations for coffee alternatives and is primarily attributed to Maillard and caramelization reactions. However, the significant color variation among beverages at this roasting level may influence consumer perception and acceptance due to visual differences in the final product.

### 3.2. Near-Infrared Spectroscopy (NIR) Analysis

**Figure 2:**
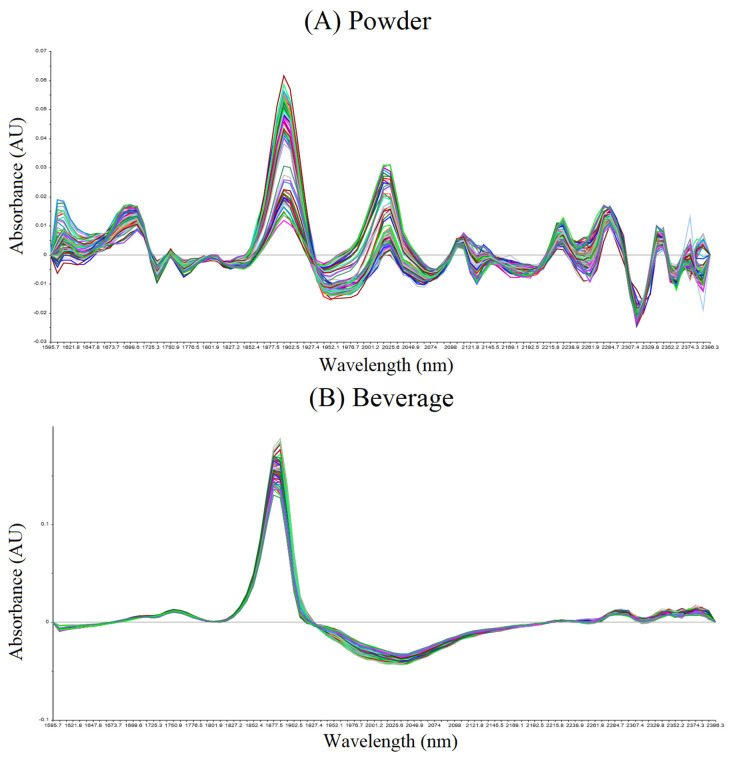
Savitzky−Golay first derivative near-infrared (NIR) spectra (1596–2396 nm) of date seed samples in powder form (**A**) and beverage form (**B**). Spectral curves of different colors represent individual replicate measurements.

To further understand the chemical basis for the observed physicochemical differences, NIR was employed. The first derivative spectra of the NIR absorbance scans for roasted date seed powder and beverage samples are presented in Figure 2. The major absorbance peaks for the powder samples ranged from 1673 to 2396 nm, whereas the beverage samples exhibited peak regions between 1720 and 1927 nm and 2238–2396 nm. These spectral features correspond to the vibrational overtones and combination bands of various chemical groups associated with compounds such as water, lipids, carbohydrates, and volatile constituents. Based on the assignment of these NIR absorption peaks, the following compounds were tentatively identified: aliphatic hydrocarbons (1730 and 2368 nm), alcohols (1737 nm), aromatic hydrocarbons (1775 nm), sucrose (2306–2312 nm), aryl compounds (1694 and 2153–2172 nm), cellulose (1825 and 2275 nm), lipids (2315 nm), and polyamides (2217 nm), as reported by [21]. The NIR spectral profiles of both date seed powders and beverages were consistent with findings from previous studies on coffee by Wu, et al. [22], highlighting the similarity in compositional and structural characteristics between roasted date seeds and traditional coffee products.

### 3.3. Electronic Nose Outputs

**Figure 3:**
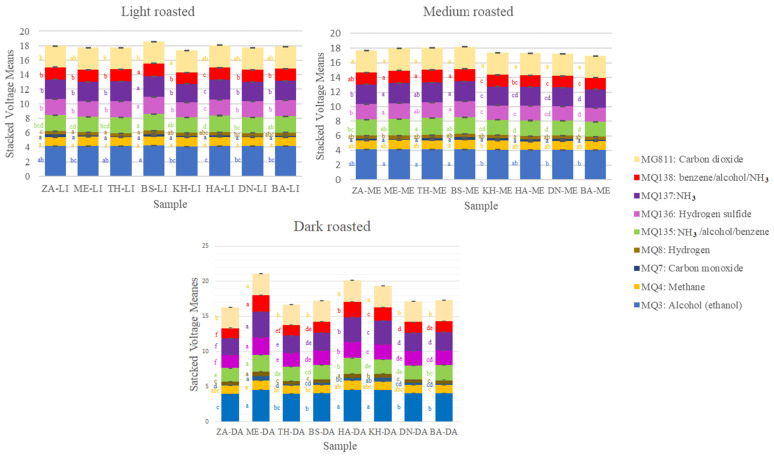
Stacked mean voltage of the nine different e-nose gas sensors among date seed beverages produced from eight cultivars of date seeds at three roasting levels. Differences are compared for each sensor among samples (bar colors). Different letters (^a–f^) denote statistically significant differences among cultivar means (*p* < 0.05), as determined by one-way analysis of variance (ANOVA) followed by Tukey’s post hoc test. For sample abbreviations, refer to Table 1.

The volatile compound profiles of date seed beverage brewed from eight cultivars of date seed at three roasting levels are shown in Figure 3. There were no significant differences in the total voltage responses across all sensors among the eight cultivars at the light and medium roasting levels. However, at the dark roasting level, beverages made from Medjool, Halawi, and Khadrawy cultivars had significantly higher sensor responses compared to the others. Among the sensors, the highest voltage was recorded by the MQ3 sensor, which is primarily sensitive to ethanol, followed by the MQ137 sensor, which detects NH_3_. These elevated responses suggest a greater release of ethanol and NH_3_-related volatiles in these particular cultivars after dark roasting, potentially due to increased degradation or transformation of organic compounds at higher roasting temperatures.

During the ripening and storage process of dates, microbial activity can act on the polysaccharides in the fruit, leading to the formation of alcohols and various organic acids, a phenomenon also observed in coffee cherries [23]. These fermentation by-products may become integrated into the cellular matrix of the date seed and subsequently be released during the brewing process. Upon roasting, date seeds emit a range of volatile organic compounds, including aldehydes, ketones, and nitrogen-containing compounds [24]. Some of these volatiles are likely responsible for fluctuations observed in the NH_3_-sensitive sensors. In this study, the Medjool cultivar showed notably higher NH_3_ sensor readings following both medium and dark roasting, suggesting an increased release of nitrogenous volatiles. Further, at the dark roasting level, the Medjool date seed beverage exhibited a significantly elevated response in the MQ138 sensor, which is sensitive to benzene and other aromatic compounds. This agrees with the findings of Farag et al. [24], who reported the presence of benzene derivatives in roasted date seed beverages.

### 3.4. Identification and Quantification of Volatile Compounds in Date Seed Beverage

The concentrations of 25 volatile compounds identified in date seed beverages made from eight cultivars at three roasting levels are presented in Table 4. The data showed significant variations in volatile profiles influenced by roasting level and cultivar differences. The results revealed significant variations in volatile profiles influenced by both roasting level and cultivar. Overall, the total volatile content tended to decrease with increasing roasting intensity, particularly for compound classes such as pyrazines and acids. In contrast, certain furanic compounds (most notably furfural) remained relatively stable across roasting levels. Among the cultivars, Bau Strami consistently exhibited the highest concentrations of key aroma-active compounds, including 2-methylpyrazine (1.76 μg/g in light roasting; 0.66 μg/g in medium roasting; 0.61 μg/g in dark roasting) and phenylethyl alcohol (0.11 μg/g in light roasting; 0.03 μg/g in medium roasting; 0.06 μg/g in dark roasting). These results may suggest the potential suitability of Bau Strami for developing a rich, coffee-like aroma profile, making it a promising candidate as a coffee alternative.

Furfural, an important furanic compound contributing to sweet and caramel-like aromas, also demonstrated cultivar-dependent trends. The Halawi cultivar showed the highest furfural concentrations at light and medium roasting levels (13.98 μg/g and 8.67 μg/g, respectively), while the Thoory cultivar exhibited the highest concentration at the dark roasting level (8.81 μg/g). These findings agree with established Maillard reaction pathways, where early stages of roasting promote the formation of pyrazines, while prolonged thermal exposure leads to their degradation and the concurrent accumulation of more thermally stable compounds such as furans, which is a trend also observed in coffee roasting studies [25,26].

Acetic acid concentrations showed a progressive decline with increasing roasting intensity, decreasing from an average of 7.14 μg/g in light-roasted samples to 3.45 μg/g in dark-roasted samples, likely due to thermal decomposition. Despite this reduction, acetic acid remained one of the most abundant volatile compounds even at the dark roasting stage. According to Diviš et al. [27], its formation is primarily attributed to the thermal degradation of saccharides (in particular sucrose and fructose) during the roasting process. This observation is consistent with findings from thermal degradation studies of carbohydrate-rich matrices, where hexose and pentose sugars undergo dehydration and fragmentation reactions that generate various organic acids, with acetic acid being a predominant byproduct [28].

The persistence of acetic acid at elevated roasting temperatures suggests a degree of thermal stability relative to other volatile compounds. Its continued presence contributes to the sour or overripe fruit-like aroma often perceived in roasted date seed beverages. This trend was most apparent in the Zahidi cultivar, where acetic acid concentrations declined from 8.42 μg/g in light-roasted samples to 2.97 μg/g in dark-roasted samples. A similar degradation pattern was observed for pyrazines. For example, 2,5-dimethylpyrazine showed an approximate 50% reduction in concentration from light to dark roasting across most cultivars. However, the Medjool cultivar demonstrated a significant retention of pyrazine compounds, particularly under medium-roasting conditions. This may suggest a possible cultivar-specific thermal stability or protective matrix effect that limits degradation of these aroma-contributing volatiles.

The persistence of furanic compounds, particularly furfural, contrasts with the general decline in volatile compounds observed with increasing roasting intensity. Although most volatile compounds decreased significantly as roasting intensity progressed, furfural concentrations remained relatively stable, exhibiting only a 20–30% reduction from light to dark roasting across most cultivars. This relative stability may be attributed to the continuous degradation of sugars, particularly pentoses, which continue to produce furfural even at elevated temperatures [29]. The Thoory cultivar demonstrated especially high furfural level, maintaining concentrations above 8 μg/g even at the dark roasting level. This trend mirrors observations in coffee roasting, where furfural functions as a key marker of caramelization and contributes to sweet, burnt-sugar aroma notes [30]. The ability of furfural to persist under high thermal conditions highlights its potential importance in contributing to the aroma complexity and sensory appeal of date seed beverages, particularly as a coffee alternative.

Minor volatile components showed more complex and non-linear patterns across roasting levels. For example, phenylethyl alcohol concentrations in the Bau Strami cultivar decreased from light to medium roasting, followed by a rebound at the dark roasting level. This irregular trend suggests the involvement of multiple, potentially competing chemical pathways, including both thermal degradation and formation from phenolic precursors. A similar pattern was observed for maltol, a compound known for its sweet, caramel-like aroma. Maltol concentrations peaked in medium-roasted samples before declining in dark-roasted samples, indicating that its formation is favored within a specific temperature range. These irregular variations highlight the intricate nature of aroma compound development during the roasting of date seeds. They emphasize the importance of precise temperature control and roasting optimization in order to maximize desirable flavor compounds while minimizing degradation. This control is essential for producing consistent and appealing sensory profiles, particularly when positioning date seed beverages as high-quality coffee alternatives.

The comparative analysis of cultivar performance revealed three distinct groups based on their volatile compound profiles. The Bau Strami and Medjool cultivars were identified as pyrazine-dominant, making them particularly well-suited for developing coffee-like roasted aromas. In contrast, the Halawi and Thoory cultivars demonstrated higher levels of furanic compounds, which are associated with sweet, caramel-like aromatic notes. The remaining cultivars (i.e., Zahidi, Deglet nour, Khadrawy, and Barhee) showed more complex and mixed volatile profiles, suggesting a broader range of aroma characteristics. These differences are likely attributable to intrinsic variations in the chemical composition of each cultivar, particularly in the ratios of reducing sugars to free amino acids, which play a critical role in driving Maillard reaction pathways [31]. The findings of this study highlight the potential of cultivar selection as a tool to modulate and optimize the aroma profiles of roasted date seed beverages, thereby enhancing their sensory appeal and positioning them as viable and attractive coffee alternatives.

**Table 4:** Concentrations of volatile compounds identified in date seed beverages by HS-SPME-GC-MS.

No.	Compound Name	Molecular Formula	Aroma	RT * (min)	Concentration (μg/g)							
					Light Roasting							
					ZA-LI	ME-LI	DN-LI	**TH-LI**	**HA-LI**	**BA-LI**	**KH-LI**	**BS-LI**
1	2-methyltetrahydrofuran-3-one	C_5_H_8_O_2_	Sweet/bready/buttery	14.64	0.19	0.13	0.15	0.24	0.21	0.16	0.15	0.15
2	2-Methylpyrazine	C_5_H_6_N_2_	Nutty/cocoa/roasted	14.73	0.59	0.72	0.51	1.03	0.95	1.10	0.35	1.76
3	Acetoin	C_4_H_8_O_2_	Sweet/buttery/creamy	15.29	0.29	0.39	0.17	0.25	0.89	1.02	0.71	0.39
4	2,5-Dimethylpyrazine	C_6_H_8_N_2_	Nutty/peanut/musty/earthy	16.54	0.05	0.09	0.05	0.10	0.10	0.11	0.03	0.24
5	2,6-Dimethylpyrazine	C_6_H_8_N_2_	Chocolate/nutty/roasted	16.72	0.13	0.16	0.12	0.24	0.24	0.26	0.10	0.49
6	2-Ethylpyrazine	C_6_H_8_N_2_	Nutty/roasted/cocoa/coffee	16.89	0.09	0.12	0.08	0.16	0.13	0.17	0.06	0.31
7	2,3-dimethylpyrazine	C_6_H_8_N_2_	Nutty/cocoa/coffee/walnut	17.26	0.03	0.03	0.02	0.03	0.05	0.07	0.05	0.10
8	2-Ethyl-6-methylpyrazine	C_7_H_10_N_2_	Roasted potato	18.47	0.05	0.07	0.05	0.10	0.08	0.11	0.05	0.21
9	Acetic acid	C_2_H_4_O_2_	Sour/overripe fruit	20.28	8.42	5.93	8.61	8.76	8.09	6.82	3.25	7.21
10	Furfural	C_5_H_4_O_2_	Sweet/woody/bready/caramel	20.64	11.16	5.67	10.18	12.84	13.98	7.72	5.91	4.49
11	2-Furyl methyl ketone	C_6_H_6_O_2_	Sweet/balsamic/almond/cocoa	21.83	1.92	1.46	1.57	2.67	2.30	1.63	0.92	2.05
12	2-Acetoxymethylfuran	C_7_H_8_O_3_	Sweet/fruity/banana/horseradish	22.76	0.38	0.21	0.26	0.38	0.20	0.23	0.22	0.30
13	2,3-Butanediol	C_4_H_10_O_2_	Fruity/creamy/buttery	22.93	0.04	0.69	0.03	0.02	0.19	0.42	0.16	1.45
14	2-Furanmethanol	C_5_H_6_O_2_	Sweet/bready/coffee	26.08	0.12	0.14	0.10	0.14	0.11	0.14	0.03	0.17
15	2(5H)-Furanone	C_4_H_4_O_2_	Buttery	28.33	0.24	0.28	0.20	0.36	0.29	0.29	0.13	0.46
16	Nonanal	C_9_H_18_O	Rose/orris/orange peel/fatty	18.66	0.02	0.02	0.04	0.04	0.04	0.02	0.01	0.01
17	5-Methylfurfuryl alcohol	C_6_H_8_O_2_	Caramellic/roasted/sweet/nutty	27.66	0.02	0.04	0.01	0.02	0.02	0.03	-	0.06
18	2,3,5,6-Tetramethylpyrazine	C_8_H_12_N_2_	Nutty/musty/chocolate/coffee	21.06	-	-	-	-	-	0.03	0.02	0.03
19	Ethyl octanoate	C_10_H_20_O_2_	Fruity/winey/waxy/sweet	19.93	-	0.01	-	-	-	-	-	0.05
20	2-Methoxy-4-vinylphenol	C_7_H_8_O_2_	Spicy/peppery/smoky/woody	30.93	0.02	0.01	0.01	0.01	0.01	0.02	0.03	0.02
21	Benzaldehyde	C_7_H_6_O	Sharp/bitter/almond	22.30	0.06	0.03	0.03	0.05	0.05	0.03	0.02	0.03
22	Dimethyl trisulfide	C_2_H_6_S_3_	Sulfurous/cooked onion/savory	18.18	0.01	0.01	0.01	0.01	-	0.01	-	0.01
23	Styrene	C_8_H_8_	Sweet/balsamic/floral/plastic	14.38	0.03	0.01	0.02	0.02	0.02	0.02	0.01	0.02
24	Maltol	C_6_H_6_O_3_	Sweet/caramellic/bread baked	33.36	0.11	0.13	0.08	0.21	0.13	0.12	0.04	0.34
25	Phenylethyl Alcohol	C_8_H_10_O	Floral/rose	32.18	0.01	0.04	0.01	0.02	0.02	0.03	0.01	0.11
**No.**	**Compound Name**	**Molecular Formula**	**Aroma**	RT * (min)	**Concentration (μg/g)**							
					**Medium Roasting**							
					**ZA-ME**	**ME-ME**	**DN-ME**	**TH-ME**	**HA-ME**	**BA-ME**	**KH-ME**	**BS-ME**
1	2-methyltetrahydrofuran-3-one	C_5_H_8_O_2_	Sweet/bready/buttery	14.64	0.13	0.14	0.11	0.16	0.13	0.18	0.12	0.18
2	2-Methylpyrazine	C_5_H_6_N_2_	Nutty/cocoa/roasted	14.73	0.20	0.33	0.17	0.23	0.22	0.46	0.18	0.66
3	Acetoin	C_4_H_8_O_2_	Sweet/buttery/creamy	15.29	0.15	0.16	0.07	0.09	0.94	0.86	1.22	0.20
4	2,5-Dimethylpyrazine	C_6_H_8_N_2_	Nutty/peanut/musty/earthy	16.54	0.02	0.03	0.02	0.02	0.02	0.05	0.01	0.07
5	2,6-Dimethylpyrazine	C_6_H_8_N_2_	Chocolate/nutty/roasted	16.72	0.05	0.08	0.05	0.07	0.06	0.13	0.05	0.20
6	2-Ethylpyrazine	C_6_H_8_N_2_	Nutty/roasted/cocoa/coffee	16.89	0.04	0.06	0.03	0.04	0.03	0.08	0.03	0.13
7	2,3-dimethylpyrazine	C_6_H_8_N_2_	Nutty/cocoa/coffee/walnut	17.26	0.01	0.02	-	0.01	0.02	0.05	0.02	0.04
8	2-Ethyl-6-methylpyrazine	C_7_H_10_N_2_	Roasted potato	18.47	0.03	0.04	0.02	0.03	0.03	0.06	0.02	0.11
9	Acetic acid	C_2_H_4_O_2_	Sour/overripe fruit	20.28	4.21	4.91	3.97	4.43	4.05	5.48	3.57	5.55
10	Furfural	C_5_H_4_O_2_	Sweet/woody/bready/caramellic	20.64	7.11	6.71	7.30	8.71	8.67	7.50	6.36	4.79
11	2-Furyl methyl ketone	C_6_H_6_O_2_	Sweet/balsamic/almond/cocoa	21.83	0.94	1.04	0.78	1.12	0.94	1.23	0.84	1.28
12	2-Acetoxymethylfuran	C_7_H_8_O_3_	Sweet/fruity/banana/horseradish	22.76	0.27	0.21	0.17	0.25	0.13	0.24	0.18	0.28
13	2,3-Butanediol	C_4_H_10_O_2_	Fruity/creamy/buttery	22.93	0.02	0.22	0.03	0.01	0.24	0.21	0.08	0.74
14	2-Furanmethanol	C_5_H_6_O_2_	Sweet/bready/coffee	26.08	0.03	0.05	0.03	0.03	0.03	0.04	0.03	0.05
15	2(5H)-Furanone	C_4_H_4_O_2_	Buttery	28.33	0.09	0.13	0.08	0.12	0.12	0.15	0.08	0.19
16	Nonanal	C_9_H_18_O	Rose/orris/orange peel/fatty	18.66	0.01	0.01	0.02	0.01	0.03	0.01	-	0.01
17	5-Methylfurfuryl alcohol	C_6_H_8_O_2_	Caramellic/roasted/sweet/nutty	27.66	-	-	-	-	-	-	-	-
18	2,3,5,6-Tetramethylpyrazine	C_8_H_12_N_2_	Nutty/musty/chocolate/coffee	21.06	-	-	-	-	-	0.02	-	0.01
19	Ethyl octanoate	C_10_H_20_O_2_	Fruity/winey/waxy/sweet	19.93	-	0.02	-	-	-	-	-	0.05
20	2-Methoxy-4-vinylphenol	C_7_H_8_O_2_	Spicy/peppery/smoky/woody	30.93	0.01	0.02	0.01	0.01	0.01	0.02	0.01	0.01
21	Benzaldehyde	C_7_H_6_O	Sharp/bitter/almond	22.30	0.03	0.02	0.02	0.02	0.03	0.03	0.03	0.02
22	Dimethyl trisulfide	C_2_H_6_S_3_	Sulfurous/cooked onion/savory	18.18	-	0.01	-	-	-	-	-	0.01
23	Styrene	C_8_H_8_	Sweet/balsamic/floral/plastic	14.38	0.02	0.03	0.01	0.01	0.02	0.02	0.01	0.02
24	Maltol	C_6_H_6_O_3_	Sweet/caramellic/bread baked	33.36	0.03	0.04	0.02	0.04	0.04	0.05	0.02	0.07
25	Phenylethyl Alcohol	C_8_H_10_O	Floral/rose	32.18	0.01	0.03	0.01	0.01	0.01	0.01	0.01	0.03
**No.**	**Compound Name**	**Molecular Formula**	**Aroma**	RT * (min)	**Concentration (μg/g)**							
					**Dark Roasting**							
					**ZA-DA**	**ME-DA**	**DN-DA**	**TH-DA**	**HA-DA**	**BA-DA**	**KH-DA**	**BS-DA**
1	2-methyltetrahydrofuran-3-one	C_5_H_8_O_2_	Sweet/bready/buttery	14.64	0.11	0.19	0.11	0.15	0.14	0.15	0.11	0.15
2	2-Methylpyrazine	C_5_H_6_N_2_	Nutty/cocoa/roasted	14.73	0.14	0.28	0.14	0.19	0.19	0.35	0.14	0.61
3	Acetoin	C_4_H_8_O_2_	Sweet/buttery/creamy	15.29	0.12	0.15	0.07	0.09	0.59	0.71	0.73	0.14
4	2,5-Dimethylpyrazine	C_6_H_8_N_2_	Nutty/peanut/musty/earthy	16.54	0.01	0.04	0.04	0.02	0.02	0.03	0.04	0.06
5	2,6-Dimethylpyrazine	C_6_H_8_N_2_	Chocolate/nutty/roasted	16.72	0.04	0.09	0.04	0.05	0.05	0.10	0.04	0.19
6	2-Ethylpyrazine	C_6_H_8_N_2_	Nutty/roasted/cocoa/coffee	16.89	0.02	0.05	0.02	0.04	0.03	0.06	0.02	0.11
7	2,3-dimethylpyrazine	C_6_H_8_N_2_	Nutty/cocoa/coffee/walnut	17.26	0.01	0.02	0.01	0.01	0.02	0.05	0.02	0.04
8	2-Ethyl-6-methylpyrazine	C_7_H_10_N_2_	Roasted potato	18.47	0.02	0.04	0.02	0.02	0.02	0.05	0.02	0.10
9	Acetic acid	C_2_H_4_O_2_	Sour/overripe fruit	20.28	2.97	3.71	3.78	3.58	3.38	3.25	3.21	3.73
10	Furfural	C_5_H_4_O_2_	Sweet/woody/bready/caramellic	20.64	6.30	5.91	7.68	8.81	8.57	5.91	7.54	4.43
11	2-Furyl methyl ketone	C_6_H_6_O_2_	Sweet/balsamic/almond/cocoa	21.83	0.74	0.91	0.82	1.00	0.90	0.92	0.94	1.18
12	2-Acetoxymethylfuran	C_7_H_8_O_3_	Sweet/fruity/banana/horseradish	22.76	0.27	0.36	0.24	0.27	0.17	0.22	0.27	0.31
13	2,3-Butanediol	C_4_H_10_O_2_	Fruity/creamy/buttery	22.93	0.02	0.28	0.02	-	0.13	0.16	0.06	0.64
14	2-Furanmethanol	C_5_H_6_O_2_	Sweet/bready/coffee	26.08	0.02	0.03	0.02	0.03	0.02	0.03	0.02	0.04
15	2(5H)-Furanone	C_4_H_4_O_2_	Buttery	28.33	0.10	0.12	0.11	0.14	0.14	0.13	0.11	0.19
16	Nonanal	C_9_H_18_O	Rose/orris/orange peel/fatty	18.66	0.01	0.01	0.01	0.01	0.02	0.01	0.01	0.01
17	5-Methylfurfuryl alcohol	C_6_H_8_O_2_	Caramellic/roasted/sweet/nutty	27.66	-	-	-	-	-	-	-	-
18	2,3,5,6-Tetramethylpyrazine	C_8_H_12_N_2_	Nutty/musty/chocolate/coffee	21.06	-	-	-	-	-	0.02	-	-
19	Ethyl octanoate	C_10_H_20_O_2_	Fruity/winey/waxy/sweet	19.93	-	0.01	-	-	-	-	-	0.08
20	2-Methoxy-4-vinylphenol	C_7_H_8_O_2_	Spicy/peppery/smoky/woody	30.93	0.02	0.05	0.02	0.01	0.01	0.03	0.03	0.02
21	Benzaldehyde	C_7_H_6_O	Sharp/bitter/almond	22.30	0.02	0.02	0.02	0.02	0.02	0.02	0.02	0.02
22	Dimethyl trisulfide	C_2_H_6_S_3_	Sulfurous/cooked onion/savory	18.18	-	0.01	-	-	-	-	-	-
23	Styrene	C_8_H_8_	Sweet/balsamic/floral/plastic	14.38	0.01	0.01	0.01	0.01	0.01	0.01	0.01	0.01
24	Maltol	C_6_H_6_O_3_	Sweet/caramellic/bread baked	33.36	0.03	0.03	0.03	0.03	0.04	0.04	0.02	0.06
25	Phenylethyl Alcohol	C_8_H_10_O	Floral/rose	32.18	0.01	0.03	0.01	0.01	0.01	0.01	0.01	0.06

* RT: retention time; -: volatile compound not detected. Note. Aroma descriptors were compiled from The Good Scents Company [32], accessed 9 June 2025.

### 3.5. Machine Learning Modeling

As shown in Table 5, Model 1 achieved an overall classification accuracy of 99.1% in distinguishing between different types of date seed beverages using NIR absorbance values as input features. The training mean squared error (MSE) was 2.33 × 10^−10^, while the testing MSE was 0.11, indicating no evidence of underfitting or overfitting and demonstrating strong generalization to unseen data. Model 2, developed using electronic nose sensor outputs as inputs, also exhibited excellent performance, achieving an overall accuracy of 97.8%, comparable to that of Model 1. Similarly, the training MSE (1.42 × 10^−9^) was substantially lower than the testing MSE (0.11). This further confirms the absence of overfitting and suggests robust predictive capabilities. The consistent performance across both training and testing datasets, as well as the reproducibility of the results across multiple retraining attempts, reinforces the reliability and generalizability of both models for accurately classifying date seed beverage samples based on their volatile or spectral signatures.

**Figure 4:**
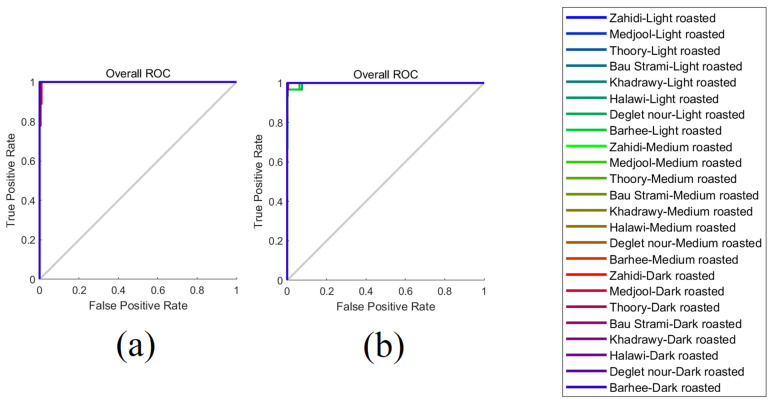
Receiver operating characteristic (ROC) curves comparing prediction performance for date seed roasting level and cultivar: (**a**) Model 1 developed using inputs of near-infrared spectral data and (**b**) Model 2 developed using the electronic nose inputs.

The receiver operating characteristic curves for Models 1 and 2 are presented in Figure 4, with both models displaying curves that closely approach the top-left corner of the plot, an indicative of high sensitivity (true positive rate) and strong overall classification performance. However, Model 2 demonstrated relatively lower sensitivity for samples derived from lightly roasted date seeds compared to those from medium and dark roasts. This reduced sensitivity may be attributed to the incomplete progression of Maillard reactions and caramelization at lower roasting temperatures, which limits the formation of volatile organic compounds detectable by the electronic nose. Therefore, the chemical profiles of lightly roasted samples exhibit fewer distinct features, leading to reduced sensor output variability and, consequently, a diminished capacity of the model to distinguish between these samples with high precision.

Table 6 summarizes the performance of Models 3 and 4, both designed to predict the concentrations of 25 volatile compounds detected by GC-MS. Model 3, which used NIR absorbance values (1596–2396 nm), achieved a correlation coefficient of *R* = 0.88, while Model 4, based on e-nose sensor outputs, achieved a slightly higher correlation of *R* = 0.90. Both models showed strong predictive performance without signs of underfitting or overfitting. The training mean squared error (MSE) values were lower than those of the testing phase, indicating good generalization to new data (Model 3: training MSE = 0.006, testing MSE = 0.240; Model 4: training MSE = 0.051, testing MSE = 0.063). These results demonstrate the effectiveness of both NIR spectroscopy and e-nose data as reliable, non-destructive tools for predicting volatile profiles in roasted date seed beverages.

**Figure 5:**
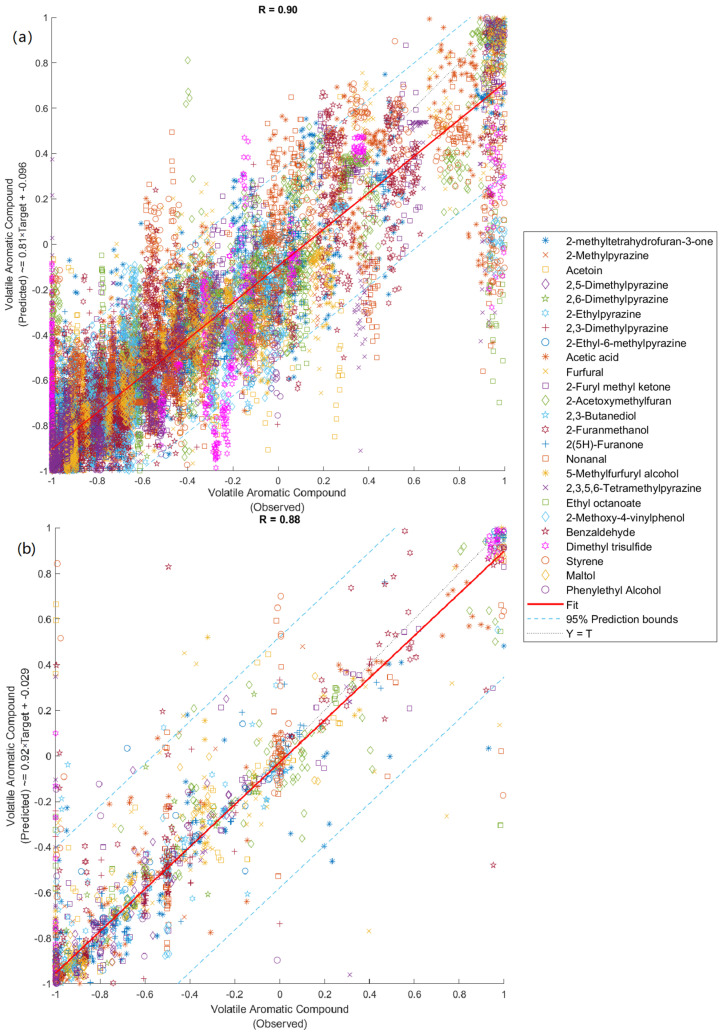
Regression Models 3 (**a**) and 4 (**b**) predicted 25 volatile aromatic compounds identified by GC-MS analysis, as shown in the scatter plots comparing observed (*x*-axis) versus predicted (*y*-axis) values. *R* = correlation coefficient.

Figure 5 illustrates the overall regression results of Models 3 and 4, each accompanied by 95% prediction bounds. Both models exhibited a strong fit, with 5% outliers identified in each case (90 out of 1800 data points). In Model 3, most outliers were associated with furfural, dimethyl trisulfide, and 2,3-butanediol. Similarly, Model 4 showed a comparable outlier rate, with deviations primarily observed in dimethyl trisulfide and 2-furanmethanol predictions. In support of these findings, prior research by Wu, Lu, Liu, Sharifi-Rad and Suleria [15] reported high predictive accuracy (*R* = 0.96–0.99) using ANN models based on NIR and e-nose data to estimate the concentrations of 14 volatile aromatic compounds in coffee. The consistency across studies supports the effectiveness of combining spectroscopic and sensor-based data with machine learning for volatile compound prediction in complex beverage matrices.

The developed models demonstrated that both NIR (Models 1 and 3) and e-nose systems (Models 2 and 4) effectively captured significant patterns within the input data, enabling accurate predictions of cultivar, roasting level, and volatile aromatic compound concentrations in date seed beverages. These tools allowed for the objective, rapid, and reliable assessment of key beverage quality attributes using portable, cost-effective, and user-friendly technologies. Future research could focus on developing advanced machine learning models that use e-nose outputs to predict the intensity of sensory descriptors, such as aroma, flavor, and mouthfeel. This approach would offer deeper predictive information into the relationships between product processing, chemical composition, sensory perception, and consumer acceptance, ultimately supporting the optimization and commercialization of date seed beverages as coffee alternatives.

## 4. Conclusions

This study demonstrated that roasting level and cultivar significantly influence the physicochemical and aromatic properties of date seed beverages. Key volatile compounds, such as pyrazines and furanic compounds, varied across cultivars and roasting intensities, affecting the sensory attributes of the final product. The application of NIR spectroscopy and e-nose technologies, combined with ANN models, enabled accurate prediction of volatile compound concentrations in different cultivars and roasting intensities. These findings confirm the effectiveness of low-cost, non-destructive analytical tools for quality assessment and classification. Overall, the results support the potential of date seeds as an underutilized raw material for developing coffee-like beverages and suggest that optimized roasting conditions and appropriate cultivar selection can contribute to consistent quality and broader application within the beverage industry.

## Figures and Tables

**Table 1:** Description of date seed beverage samples used in this study, including cultivar, roasting level, and sample abbreviation.

Cultivar	Light Roasted (LI)	Medium Roasted (ME)	Dark Roasted (DA)
**Zahidi**	ZA-LI	ZA-ME	ZA-DA
**Medjool**	ME-LI	ME-ME	ME-DA
**Deglet nour**	DN-LI	DN-ME	DN-DA
**Thoory**	TH-LI	TH-ME	TH-DA
**Halawi**	HA-LI	HA-ME	HA-DA
**Barhee**	BA-LI	BA-ME	BA-DA
**Khadrawy**	KH-LI	KH-ME	KH-DA
**Bau Strami**	BS-LI	BS-ME	BS-DA

**Table 2:** The pH of different roasting levels and cultivars of date seeds.

Sample Name	Zahidi	Medjool	Deglet Nour	Thoory	Halawi	Barhee	Khadrawy	Bau Strami	
Roasting Level	pH								Average
Light	**ZA-LI**	**ME-LI**	**DN-LI**	**TH-LI**	**HA-LI**	**BA-LI**	**KH-LI**	**BS-LI**	
	6.22 ± 0.01 ^aA^	5.72 ± 0.01 ^cA^	5.38 ± 0.05 ^dA^	5.89 ± 0.03 ^bA^	5.90 ± 0.03 ^bA^	5.95 ± 0.03 ^bA^	5.49 ± 0.05 ^cA^	5.95 ± 0.02 ^bA^	5.95 ± 0.05
Medium	**ZA-ME**	**ME-ME**	**DN-ME**	**TH-ME**	**HA-ME**	**BA-ME**	**KH-ME**	**BS-ME**	
	4.30 ± 0.02 ^aB^	4.37 ± 0.02 ^aB^	3.71 ± 0.01 ^cB^	3.77 ± 0.02 ^cB^	4.03 ± 0.02 ^bB^	4.04 ± 0.05 ^bB^	3.79 ± 0.02 ^cB^	3.94 ± 0.03 ^bB^	3.99 ± 0.05
Dark	**ZA-DA**	**ME-DA**	**DN-DA**	**TH-DA**	**HA-DA**	**BA-DA**	**KH-DA**	**BS-DA**	
	3.97 ± 0.01 ^aC^	3.25 ± 0.01 ^cC^	3.76 ± 0.06 ^abB^	3.93 ± 0.06 ^abB^	3.30 ± 0.02 ^cC^	3.87 ± 0.02 ^abC^	3.39 ± 0.09 ^cB^	3.74 ± 0.06 ^bB^	3.65 ± 0.06

The data are shown as mean ± standard error (*n* = 3); ^a,b,c,d^ indicate the means in a row, ^A,B,C^ indicate the means in a column with significant difference (*p* < 0.05) using a one-way analysis of variance (ANOVA) and Tukey’s test.

**Table 3:** Color measurements of date seed powder and beverage made from different roasting levels and cultivars of date seeds.

Sample		Light Roasted			Medium Roasted			Dark Roasted		
		L*	a*	b*	L*	a*	b*	L*	a*	b*
Powder	Zahidi	46.33 ± 1.12 ^ab^	15.07 ± 0.49 ^a^	22.17 ± 0.21 ^ab^	20.53 ± 6.70 ^a^	12.40 ± 2.72 ^a^	14.40 ± 4.46 ^a^	20.67 ± 0.83 ^ab^	10.73 ± 0.32 ^bcd^	12.67 ± 0.38 ^abc^
	Medjool	43.80 ± 7.24 ^ab^	14.73 ± 0.29 ^a^	21.30 ± 2.25 ^ab^	27.13 ± 1.89 ^a^	15.20 ± 0.92 ^a^	18.33 ± 1.04 ^a^	20.67 ± 1.86 ^ab^	13.47 ± 0.46 ^a^	14.97 ± 0.14 ^a^
	Deglet nour	45.57 ± 0.75 ^ab^	14.87 ± 0.83 ^a^	22.40 ± 0.36 ^ab^	21.03 ± 3.10 ^a^	12.17 ± 1.15 ^a^	14.47 ± 1.80 ^a^	18.03 ± 1.91 ^b^	9.37 ± 0.45 ^de^	11.20 ± 0.36 ^c^
	Thoory	51.90 ± 0.62 ^a^	14.43 ± 0.29 ^a^	23.10 ± 0.20 ^a^	22.20 ± 1.21 ^a^	12.50 ± 0.50 ^a^	14.83 ± 0.80 ^a^	18.20 ± 0.99 ^ab^	9.17 ± 0.45 ^e^	10.80 ± 0.56 ^c^
	Halawi	43.30 ± 1.49 ^ab^	13.07 ± 0.23 ^ab^	19.73 ± 0.57 ^ab^	23.40 ± 3.54 ^a^	13.37 ± 1.76 ^a^	16.10 ± 2.36 ^a^	16.87 ± 1.25 ^b^	10.83 ± 0.15 ^bcd^	11.30 ± 0.62 ^c^
	Barhee	31.23 ± 3.96 ^c^	11.43 ± 1.11 ^b^	16.13 ± 2.05 ^c^	24.73 ± 3.06 ^a^	14.10 ± 1.31 ^a^	16.87 ± 1.89 ^a^	18.97 ± 0.99 ^ab^	10.03 ± 0.49 ^cde^	11.73 ± 0.58 ^c^
	Khadrawy	39.43 ± 2.91 ^bc^	13.80 ± 0.89 ^a^	19.43 ± 1.36 ^bc^	24.80 ± 1.50 ^a^	13.53 ± 0.55 ^a^	15.90 ± 0.70 ^a^	16.87 ± 1.32 ^b^	11.27 ± 1.17 ^bc^	12.33 ± 0.99 ^bc^
	Bau Strami	46.23 ± 0.93 ^ab^	14.37 ± 0.45 ^a^	21.93 ± 0.32 ^ab^	23.67 ± 2.90 ^a^	13.70 ± 1.31 ^a^	16.53 ± 1.80 ^a^	22.83 ± 0.21 ^a^	11.80 ± 0.11 ^b^	14.67 ± 0.15 ^a^
	Average	43.48 ± 6.38	13.97 ± 1.30	20.78 ± 2.39	23.44 ± 3.51	13.37 ± 1.54	15.93 ± 2.22	19.14 ± 2.44	10.83 ± 1.43	12.46 ± 1.69
ΔE				20.62			6.08			
Beverage	Zahidi	52.67 ± 1.83 ^a^	5.13 ± 0.32 ^a^	−10.33 ± 1.01 ^a^	44.70 ± 5.09 ^b^	4.77 ± 0.32 ^a^	10.20 ± 4.10 ^ab^	48.20 ± 0.40 ^c^	3.47 ± 0.21 ^a^	6.50 ± 1.25 ^bc^
	Medjool	58.60 ± 8.79 ^a^	3.30 ± 2.08 ^ab^	−15.37 ± 7.29 ^a^	51.97 ± 1.65 ^ab^	1.33 ± 0.50 ^cd^	−5.37 ± 3.83 ^c^	31.93 ± 1.92 ^e^	2.03 ± 0.38 ^ab^	19.03 ± 1.47 ^a^
	Deglet nour	53.53 ± 1.70 ^a^	3.13 ± 0.58 ^ab^	−12.70 ± 0.80 ^a^	46.63 ± 1.41 ^ab^	2.63 ± 0.29 ^b^	8.63 ± 1.89 ^ab^	48.83 ± 0.67 ^bc^	1.50 ± 0.10 ^ab^	1.10 ± 0.10 ^c^
	Thoory	50.90 ± 4.61 ^a^	4.00 ± 0.17 ^ab^	−11.30 ± 1.21 ^a^	48.27 ± 1.32 ^ab^	1.00 ± 0.36 ^d^	3.80 ± 1.30 ^b^	55.80 ± 2.02 ^a^	0.80 ± 0.44 ^ab^	−9.07 ± 2.29 ^d^
	Halawi	54.10 ± 1.40 ^a^	2.53 ± 0.32 ^b^	−15.53 ± 1.60 ^a^	47.43 ± 2.32 ^ab^	2.17 ± 0.50 ^bc^	12.80 ± 2.79 ^a^	41.77 ± 2.15 ^d^	0.63 ± 2.75 ^ab^	11.47 ± 1.41 ^ab^
	Barhee	55.07 ± 3.27 ^a^	2.27 ± 0.29 ^b^	−18.20 ± 3.63 ^a^	46.67 ± 2.89 ^ab^	−0.77 ± 0.23 ^e^	5.80 ± 3.24 ^ab^	53.37 ± 0.93 ^ab^	−0.40 ± 0.46 ^b^	2.50 ± 1.06 ^c^
	Khadrawy	52.63 ± 4.96 ^a^	3.63 ± 0.32 ^ab^	−12.40 ± 1.75 ^a^	54.77 ± 3.25 ^a^	0.67 ± 0.50 ^d^	−9.13 ± 1.21 ^c^	41.30 ± 2.10 ^d^	1.13 ± 0.87 ^ab^	12.53 ± 1.93 ^ab^
	Bau Strami	49.47 ± 1.36 ^a^	3.47 ± 0.57 ^ab^	−11.57 ± 0.80 ^a^	46.80 ± 3.75 ^ab^	2.12 ± 0.06 ^bc^	7.87 ± 4.04 ^ab^	47.90 ± 2.01 ^c^	2.37 ± 0.31 ^ab^	9.17 ± 2.32 ^bc^
	Average	53.37 ± 4.41	3.43 ± 1.09	−13.43 ± 3.62	48.40 ± 4.02	1.74 ± 1.58	4.33 ± 7.78	46.77 ± 6.87	1.44 ± 1.44	6.65 ± 8.56
ΔE				18.51			2.86			

All values represent the average of three replicates from date seed powder and beverage with eight different cultivars and three different roasting levels. ΔE represents the color difference between the average value of two adjacent roasting levels, calculated using Equation (1). ^a–e^ indicate the mean values of different cultivars that show significant differences (*p* < 0.05) using a one-way analysis of variance (ANOVA) and Tukey’s test.

**Table 5:** Results of the classification of artificial neural network Models 1 and 2.

Stage	Samples	Accuracy	Error	Performance (MSE)
Model 1: Inputs: NIR; Targets: type of date seed beverage				
Training	151	100%	0%	2.33 × 10^−10^
Testing	65	96.9%	3.1%	0.11
Overall	216	99.1%	0.9%	-
Model 2: Inputs: electronic nose; Targets: type of date seed beverage				
Training	504	100%	0%	1.42 × 10^−9^
Testing	216	92.6%	7.4%	0.11
Overall	720	97.8%	2.2%	-

MSE: means mean squared error.

**Table 6:** The results of the artificial neural network regression Models 3 and 4.

Stage	Samples	Observations	R	Slope	Performance (MSE)
Model 3: Inputs: NIR; Targets: volatile aromatic compounds					
Training	50	1250	0.99	0.87	0.006
Testing	22	550	0.67	0.80	0.240
Overall	72	1800	0.88	0.92	-
Model 4: Inputs: electronic nose; Targets: volatile aromatic compounds					
Training	504	12,600	0.90	0.81	0.051
Testing	216	5400	0.88	0.79	0.063
Overall	720	18,000	0.90	0.81	-

R: correlation coefficient; MSE: mean squared error.

## Data Availability

The original contributions presented in this study are included in the article. Further inquiries can be directed to the corresponding author.
